# The implications of FASN in immune cell biology and related diseases

**DOI:** 10.1038/s41419-024-06463-6

**Published:** 2024-01-25

**Authors:** Yucai Xiao, Yonghong Yang, Huabao Xiong, Guanjun Dong

**Affiliations:** 1 Cheeloo College of Medicine, Shandong University, Jinan, 250012 Shandong China; 2 Institute of Immunology and Molecular Medicine, Jining Medical University, Jining, 272067 Shandong China; 3 Jining Key Laboratory of Immunology, Jining Medical University, Jining, 272067 Shandong China; 4 Medical Research Center, Affiliated Hospital of Jining Medical University, Jining, 272007 Shandong China

**Keywords:** Mechanisms of disease, Fatty acids

## Abstract

Fatty acid metabolism, particularly fatty acid synthesis, is a very important cellular physiological process in which nutrients are used for energy storage and biofilm synthesis. As a key enzyme in the fatty acid metabolism, fatty acid synthase (FASN) is receiving increasing attention. Although previous studies on FASN have mainly focused on various malignancies, many studies have recently reported that FASN regulates the survival, differentiation, and function of various immune cells, and subsequently participates in the occurrence and development of immune-related diseases. However, few studies to date systematically summarized the function and molecular mechanisms of FASN in immune cell biology and related diseases. In this review, we discuss the regulatory effect of FASN on immune cells, and the progress in research on the implications of FASN in immune-related diseases. Understanding the function of FASN in immune cell biology and related diseases can offer insights into novel treatment strategies for clinical diseases.

## Facts

- FASN, a key enzyme in fatty acid metabolism, can regulate the survival, differentiation and function of various immune cells.
- Dysregulation of FASN contributes to the occurrence and development of immune-related diseases.
- Targeting FASN has gained considerable attention as a promising therapeutic approach for immune-related diseases.

## Open Questions

- What factors are involved in regulating the expression of FASN in different immune microenvironments?
- What is the regulatory effect of FASN on immune cells and its mechanism?
- What is the role of FASN in the pathogenesis of different types of immune-associated diseases?
- How does targeted inhibition of FASN affect the incidence of immune-associated diseases?

## Introduction

**Fig. 1:**
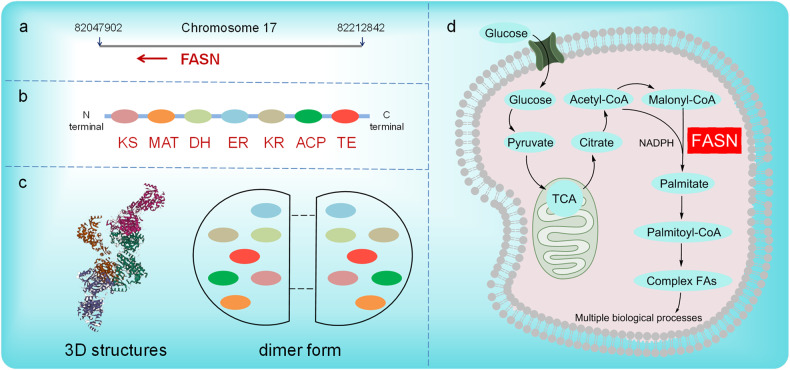
Basic information on the FASN gene and protein and the function of FASN. **a** The location of the FASN gene. **b** Generalized linear domain map of animal FASN. **c** The basic structure of the human FASN protein 3D structure (http://www.genecards.org). **d** FASN-mediated biosynthesis pathway of fatty acids. Glucose is phosphorylated to glucose-6P when ingested by cells and enters the glycolysis pathway. The resulting pyruvate enters the mitochondria and is converted to acetyl-CoA before entering the citric acid cycle. The citrate then travels through the mitochondria and is converted to acetyl-CoA by the ACL. Then, acetyl-CoA is converted to malonyl CoA. Using NADPH as a reduction cofactor, FASN catalyzes the synthesis of acetyl-CoA and malonyl-CoA palmitate and then synthesizes complex fatty acid metabolites. TCA tricarboxylic acid cycle, NADPH nicotinamide adenine dinucleotide phosphate hydrogen.

Fatty Acid Synthetase (FASN) is an essential enzyme in the de novo synthesis of endogenous long-chain fatty acids [1]. During the catalysis of FASN, acetyl CoA and malonyl CoA are used as raw materials, and the main products include palmitic acid (80%), stearic acid (10%), and myristic acid (10%) [2]. As a macromolecular multifunctional polymerase, FASN comprises seven catalytic domains, including condensation, transacylation, reduction, and dehydration [3]. In humans, the gene encoding FASN is located on chromosome 17q25; it is approximately 20 kb long, and contains 43 exons, 42 introns, 3 initiation sites, and 3 promoters. In mammals, FASN exists and functions as a dimer, with two identical peptide chains, and a molecular weight of approximately 260 kDa each. Once the dimer is depolymerized, it loses its activity [3]. The N-terminal region of FASN contains three catalytic domains (ketoacyl-synthase, dehydrase, and monoacyl/acetyltransferase), and the C-terminal region contains four domains (alcohol reductase, ketoacyl-reductase, acyl carrier protein, and thioesterase), with a core region comprising 600 amino acid residues in the middle (Fig. 1).

Under physiological conditions, human tissues mainly use exogenous fatty acids, and the synthesis level of endogenous fatty acids is low. In some pathological conditions such as tumors [4, 5], cardiovascular diseases [6, 7], inflammatory diseases [8–11], and autoimmune diseases [12, 13], FASN expression and the content of fatty acid products synthesized from FASN are abnormally changed. Since numerous studies have corroborated the vital role of FASN-mediated fatty acid synthesis in supporting cellular activities and its involvement in diverse biological processes [14], FASN has been identified as a significant contributor to the progression of numerous diseases. Consequently, targeting FASN has gained considerable attention as a promising therapeutic approach for these conditions, thus emerging as a prominent area of research interest [2].

This review provides an overview of our current understanding of the molecular mechanism of FASN expression regulation. Furthermore, we highlight the implications of FASN in immune cell biology and related diseases. By understanding these processes, future therapeutic strategies could be shaped to develop new strategies for treating immune-related diseases.

## Regulation of FASN expression and activity

### Transcription factor SREBP

Sterol regulatory element-binding proteins (SREBPs) are crucial nuclear transcription factors that participate in lipid metabolism in eukaryotic cells [15]. Extensive research has substantiated that SREBPs play a significant role in promoting the expression of FASN by recognizing binding sites at the proximal promoter of FASN [16]. Although SREBPs consist of three subtypes, SREBP-1a, SREBP-1c and SREBP-2, it has been discovered that only SREBP-1a and SREBP-1c can activate the transcription of genes involved in fatty acid and triglyceride synthesis, including FASN. In a normal physiological state, SREBPs exist in an inactive form (named pSREBP), mainly on the surface of the endoplasmic reticulum and nuclear membrane. However, under certain pathological conditions or when lipid levels are low, SREBP undergoes sequential cleavage, leading to the formation of mature SREBP. Mature SREBP is then transported into the nucleus, and promotes the transcription of various target genes implicated in adipogenesis and cholesterol biosynthesis [17].

**Fig. 2:**
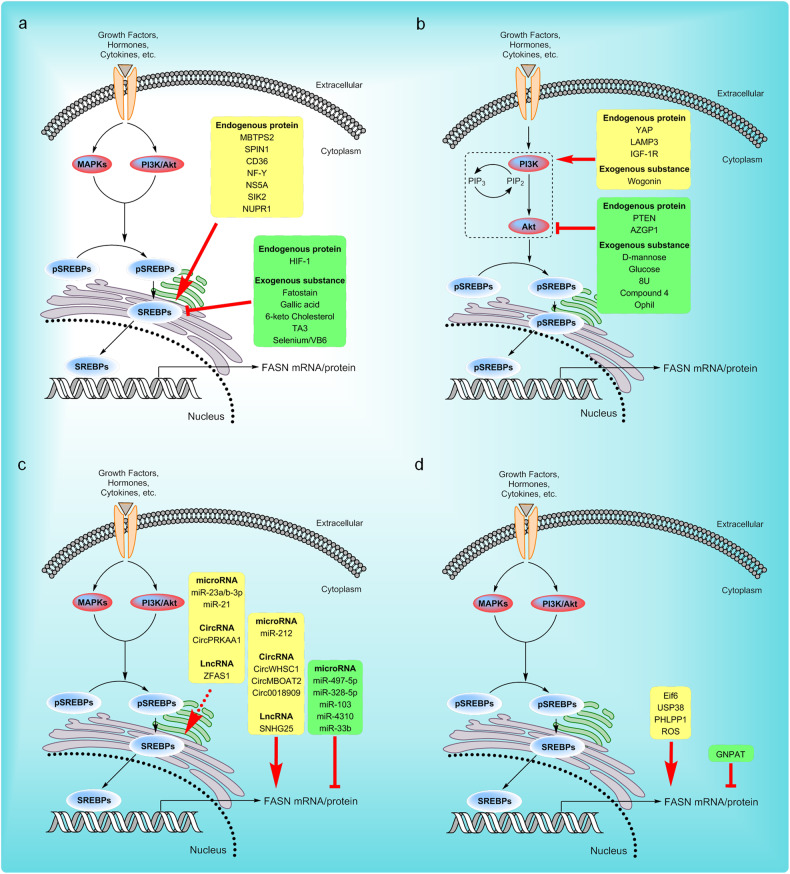
Molecular mechanism of FASN gene or protein expression regulation. **a** SREBPs play a significant role in controlling the expression of FASN by recognizing binding sites at the proximal promoter of FASN. Endogenous proteins and exogenous substances can promote or inhibit SREBP-mediated FASN transcription. **b** The PI3K-AKT signaling pathway participates in regulating the SREBP-FASN signal axis. Endogenous proteins and exogenous substances can regulate FASN expression by triggering or inhibiting the PI3K-AKT signaling pathway. **c** Noncoding RNAs can regulate FASN expression by targeting FASN or SREBPs. **d** Some proteins can directly regulate the stability of FASN at the mRNA or protein level. MBTPS2 membrane-bound transcription factor protease site 2, SPIN1 spindle protein 1, NF-Y nuclear factor Y, NS5A nonstructural protein 5A, SIK2 salt inducible kinase 2, NUPR1 nuclear protein 1, HIF1 hypoxia-inducible factor-1, YAP Yes-associated protein, LAMP3 lysosomal associated membrane protein 3, IGF-1R insulin-like growth factor 1 receptor, PTEN phosphatase and tensin homolog deleted on chromosome ten, AZGP1 Zinc-alpha-2-glycoprotein, Eif6 eukaryotic initiation factor 6, USP38 ubiquitin carboxyl-terminal hydrolase 38, PHLPP1 PH domain leucine-rich repeat protein phosphatase 1, ROS reactive oxygen species, GNPAT glyceronephosphate Oacyltransferase.

Regulation of the SREBP-FASN axis is involved in different biological processes. Many proteins, including membrane-bound transcription factor protease site 2 (MBTPS2), CD36, spindle protein 1 (SPIN1), and so on, can promote the expression of FASN by enhancing the expression of SREBP, interacting with SREBPs, or promoting the nuclear translocation of SREBPs to participate in the regulation of biological processes mediated by fatty acid metabolism [18–23]. For example, nuclear factor Y (NF-Y) activates the transcription of SREBP-1 by directly binding to the CCAAT motif in the SREBP-1 promoter and subsequently leads to enhanced expression of SREBP-1 and FASN, consequently contributing to the progression of alcoholic liver disease [24]. Targeted inhibition of SREBPs can consistently inhibit FASN expression and FASN-mediated fatty acid synthesis [25–28]. For instance, in high-fat diet-induced hyperlipidemic rats, the combined supplementation of selenium and vitamin B6 inhibits FASN expression by blocking the SIRT1/SREBP-1c signaling axis, and this intervention demonstrates an improvement in dyslipidemia and fatty liver syndrome [29]. In conclusion, all these studies indicate that SREBPs can indeed regulate the expression of FASN (Fig. 2a and Table 1).

**Table 1:** Regulation of FASN expression and activity.

Protein, inhibitor or ncRNA	Target	Effect on FASN	Mechanism and biology function	Reference
MBTPS2	SREBP	Upregulate	Upregulates FASN expression and promotes lipogenesis synthesis by enhancing the expression of SREBP	[18]
SPIN1	SREBP	Upregulate	Activates FASN transcription by interacting with SREBP-1c, thereby promoting hepatocellular cancer cell growth	[19]
CD36	SREBP	Upregulate	Promotes the expression of SREBP1 and its inducer gene FASN, ultimately leading to HFD-induced hepatic steatosis	[20]
HIF-1	SREBP	Downregulate	HIF-1 deletion promotes the nuclear translocation of SREBP-1c, induce FASN expression, and promote FASN-mediated lipid accumulation, thereby leading to impairment of host protection against leishmaniasis in myeloid cells	[21]
NS5A	SREBP	Upregulate	Facilitates SREBP-1c-mediated FASN expression, thereby promoting hepatic lipid accumulation	[22]
NUPR1	SREBP	Upregulate	Interacts with SREBP-1 to upregulate the expression of FASN, leading to lipid accumulation	[23]
NF-Y	SREBP	Upregulate	Directly binds to the CCAAT regulatory motif in the promoter, activating the transcription of SREBP-1, enhances the expression of SREBP-1 and FASN, consequently contributing to the progression of alcoholic liver disease	[24]
Fatostatin	SREBP	Downregulate	Play an anti-prostate cancer role by inhibiting the expression of FASN	[25]
Gallic acid	SREBP	Downregulate	Impairs fructose-driven de novo lipogenesis and ameliorates hepatic steatosis by inhibiting the SREBP-1/FASN cascade	[26]
6-Keto cholesterol	SREBP	Downregulate	Inhibits FASN expression by downregulating SREBP expression, thereby inhibiting lipid accumulation in HepG2 cells	[27]
Timosaponin A3	SREBP	Downregulate	Controls the growth of BxPC-3 cells by downregulating FASN expression through the inhibition of SREBP-1	[28]
Selenium and vitamin B6	SREBP	Downregulate	Inhibits FASN expression by blocking the SIRT1/SREBP-1c axis, demonstrating an improvement in dyslipidemia and fatty liver syndrome	[29]
YAP	PI3K/Akt	Upregulate	Facilitates FASN-dependent lipogenesis by engaging the rapamycin (mTOR) complex 1 (mTORC1) signaling pathway	[31]
D-Mannose	PI3K/Akt	Downregulate	Modulates lipid metabolism via the PI3K/Akt/FASN pathway, exhibiting a protective role against hepatic steatosis	[32]
Glucose	PI3K/Akt	Downregulate	Inhibits FASN expression in Schwann cells through the blockade of PI3K/Akt pathway, thereby contributing to the development of diabetic peripheral neuropathy.	[33]
LAMP3	PI3K/Akt	Upregulate	Triggers Akt activation, resulting in the upregulation of FASN expression in HepG2 cells.	[34]
SIK2	SREBP	Upregulate	Upregulates the expression of SREBP-1c, promoting the transcription of FASN and the synthesis of fatty acids, ultimately contributing to the development of ovarian cancer (OC)	[35]
AZGP1	PI3K/Akt	Downregulate	Suppresses the activity of colorectal cancer cells by modulating FASN through the mTOR pathway	[36]
IGF-1R	PI3K/Akt	Upregulate	Upregulates FASN in breast cancer, consequently promoting breast cancer incidence	[37]
PTEN	PI3K/Akt	Downregulate	Targets PIP3 to block AKT activation, thereby blocking the PI3K-AKT pathway and inhibiting FASN expression	[38]
Compound 8u	PI3K/Akt	Downregulate	Blocks the PI3K/Akt pathway, resulting in reduced FASN protein expression, hampers cell invasion and metastasis	[39]
Compound 4	PI3K/Akt	Downregulate	Suppresses ovarian cancer incidence by inhibiting the PI3K/AKT pathway and downregulating FASN expression	[40]
Wogonin	PI3K/Akt	Upregulate	Actives AKT and SREBP1 nuclear accumulation, significantly increase FASN expression and regulate fatty acid metabolism	[41]
Orlistat	PI3K/Akt	Downregulate	Inhibits FASN expression by regulating AKT pathway, thereby exerting anti-lipogenesis and anti-proliferative effects	[42]
miR-497-5p	FASN	Downregulate	Target FASN 3’-UTR and inhibit FASN expression, thus restraining CC development	[45]
miR-328-5p	FASN	Downregulate	Target FASN 3’-UTR and inhibit FASN expression, thus promoting hMSCs adipogenic differentiation	[46]
miR-103	FASN	Downregulate	Target FASN 3’-UTR and inhibit FASN expression, thus alleviating the pathogenesis of NAFLD	[47]
miR-4310	FASN	Downregulate	Target FASN 3’-UTR and inhibit FASN expression, thus inhibiting HCC cell proliferation, migration, and invasion in vitro and suppresses HCC tumor growth and metastasis	[48]
miR-33b	TAK1	Downregulate	Directly targets TAK1, leading to the inhibition of FASN activity and modulation of lipid metabolism	[49]
miRNA-212	SIRT2	Upregulate	Promotes FASN expression by targeting SIRT2, thereby promoting lipogenesis in mammary epithelial cell lines	[50]
lncSNHG25	FASN	Upregulate	Regulates FASN expression through miR-497–5p, thereby promoting the malignancy of endometrial cancer	[51]
CircWHSC1	FASN	Upregulate	Enhances FASN expression through miR-195–5p, thereby promoting breast cancer progression	[52]
circMBOAT2	FASN	Upregulate	Promotes lipid metabolism by stabilizing PTBP1 to promote the cytoplasmic output of FASN mRNA	[53]
Circ_0018909	FASN	Upregulate	Tromotes the expression of FASN by regulating miR-545–3p, thereby promoting cell growth, migration, invasion, EMT, and elevated the number of apoptotic cells in pancreatic cancer cells, as well as tumor growth	[54]
miR-23a/b-3p	SREBP-1c	Upregulate	Enhances mRNA stability by binding to the 5ʹ-UTR of both SREBP-1c and FASN mRNA, thereby facilitating triglyceride accumulation in hepatocytes	[55]
miR-21	SREBP-1	Upregulate	Activates the IRS1/SREBP-1 axis, leading to the upregulation of FASN expression and disease progression	[56]
lncZFAS1	SREBP-1	Upregulate	Interacts with PABP2, promotes the stabilization of SREBP-1 mRNA, elevates the expression of SREBP-1 and FASN, thereby promoting lipid accumulation in colorectal cancer	[57]
Circ PRKAA1	SREBP-1	Upregulate	Enhances the stability of SREBP-1 and selectively binds to the promoter region of the FASN gene, thereby increasing fatty acid synthesis to promote cancer growth	[58]
Eif6	FASN	Upregulate	Promotes atherosclerosis development by upregulating FASN expression and other fatty acid synthesis genes	[59]
USP38	FASN	Upregulate	Interacts with FASN, thereby enhancing the stability of FASN protein and promoting progression of gastric cancer	[60]
PHLPP1	FASN	Upregulate	Interacts with ChREBP and enhances ChREBP recruitment to FASN promoter, subsequently promoting lipid formation	[61]
GNPAT	FASN	Downregulate	Regulates lipid metabolism and liver cancer by inhibiting FASN degradation mediated by TRIM21	[62]

### PI3K-AKT signaling pathway

Phosphatidylinositol kinase PI3K is a very important phosphatidylinositol 3-kinase that produces the second messenger phosphatidylinositol 3,4,5-triphosphate (PI-3,4,5-P3). As a critical regulatory molecule for cellular metabolism and growth, PI3K governs essential biological processes such as nutrient uptake, energy generation, cofactor production, and macromolecular biosynthesis [30]. Consequently, PI3K can be regarded as the central driving force behind cellular metabolism.

There is substantial evidence that the PI3K-AKT signaling pathway participates in many biological processes by regulating the SREBP-FASN signaling axis [31–37]. For example, PTEN, a protein phospholipid phosphatase, can target PIP3 to block AKT activation, thereby inhibiting FASN expression [38]. In addition, pharmacological inhibition of the PI3K-AKT pathway can effectively impede FASN activity or expression, and its associated fatty acid synthesis [39–41]. For instance, orlistat can inhibit FASN activity by regulating the AKT pathway, thereby exerting its anti-lipogenesis and anti-proliferative effects [42]. In conclusion, the PI3K-AKT pathway has a critical impact on the regulation of FASN expression and thereby influences multiple biological processes (Fig. 2b and Table 1).

### Noncoding RNA (ncRNA)

Noncoding RNAs, including microRNAs, long-chain noncoding RNAs, and circulating RNAs, have been shown to widely participate in various networks in which they can modulate complex molecular and cellular processes [43, 44]. Notably, it has been shown that noncoding RNAs can directly or indirectly regulate FASN expression and modulate fatty acid synthesis.

At present, the regulation of miRNA on the expression of FASN has been widely studied. On the one hand, some miRNAs, including miR-497-5p, miR-328-5p, miR-103, and miR-4310, can directly target FASN and inhibit its expression [45–48]. On the other hand, some miRNAs can regulate FASN expression indirectly. For example, miR-33b can target the transformation of growth factor β-activated kinase 1 (TAK1), leading to the inhibition of FASN activity and modulation of lipid metabolism [49]. Another miRNA, miRNA-212, regulates FASN expression by specifically targeting SIRT2 [50]. However, to date, there are few studies on the regulation of FASN expression by long noncoding RNAs and circulating RNAs. Small nucleolar RNA host gene 25 (SNHG25), a long-strand ncRNA, regulates FASN expression through miR-497–5p [51]. Some circRNAs can also regulate FASN expression and participate in multiple biological processes [52–54].

Notably, some noncoding RNAs can indirectly regulate FASN expression and modulate fatty acid synthesis by influencing the expression or activity of SREBPs [55–57]. For instance, the long noncoding RNA ZFAS1 interacts with polyadenylate binding protein 2 (PABP2), promoting the stabilization of SREBP-1 mRNA. Consequently, this interaction elevates the expression of SREBP-1 and its downstream target gene FASN, thereby promoting lipid accumulation in colorectal cancer (CRC) [58]. In conclusion, noncoding RNAs are also involved in regulating the expression of FASN (Fig. 2c and Table 1).

### Other proteins

In addition to the above three key molecules or signaling pathways involved in regulating FASN expression, several other proteins have also been found to be involved in regulating FASN expression, including eukaryotic initiation factor 6 (Eif6), the ubiquitin-specific protease USP38, pleckstrin homology domain leucine-rich repeat protein phosphatase 1 (PHLPP1), and glycerophospho-o-acyltransferase (GNPAT) [59–62]. These molecules can affect the stability of FASN at the mRNA or protein level and thus participate in various biological processes (Fig. 2d and Table 1).

## FASN and immune cells

### Macrophages

Macrophages, which are an important component of the innate immune system, exhibit distinct activation states and diverse characteristics and functions influenced by alterations in the tissue microenvironment. Over the past years, multiple investigations have demonstrated the significant involvement of macrophage polarization, primarily categorized as M1 and M2, in various pathophysiological processes, including inflammation, tumor development, tissue repair, and metabolism. M1 macrophages can promote inflammation, eliminate pathogenic microorganisms, and show antitumor effects; M2 macrophages exhibit regulatory functions, such as inhibiting inflammation and promoting tissue remodeling [63, 64].

**Fig. 3:**
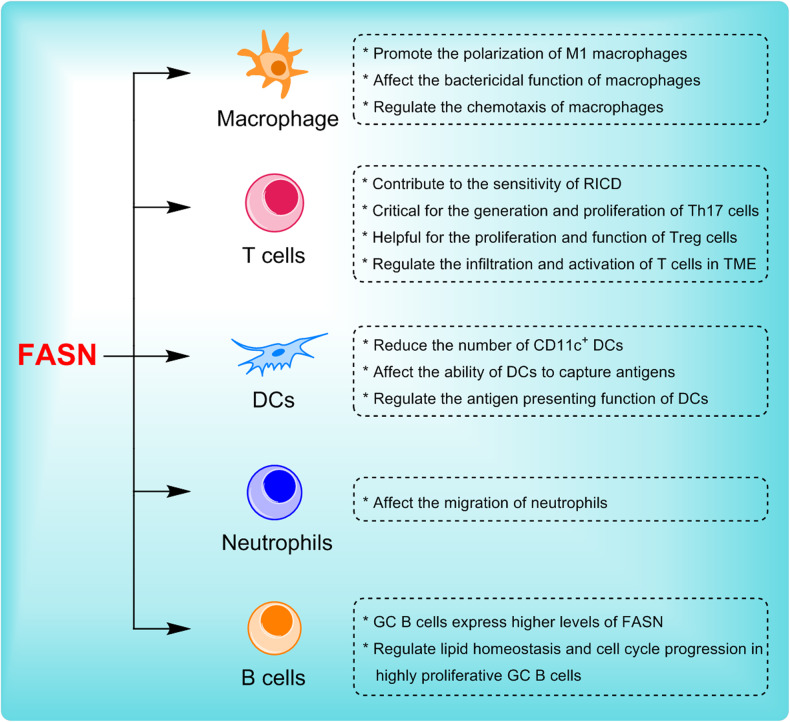
The regulatory effect of FASN on immune cells. FASN regulates the differentiation, development, chemotaxis, activation, and function of immune cells.

It has been demonstrated that FASN and FASN-mediated fatty acid synthesis play a crucial regulatory role in the polarization of M1 macrophages. The polarization of M0 macrophages to M1 macrophages is accompanied by an enhanced level of FASN expression and significant changes in FASN-mediated fatty acid metabolism [9]; high expression of FASN can significantly promote M1-type macrophage activation, while the inhibition or deletion of FASN can significantly reduce M1-type macrophage activation [9, 65]. For instance, the deletion of FASN, particularly its ketoacyl synthase domain, impairs M1 macrophage activation by reducing acetoacetyl-CoA levels and thereby obstructing cholesterol synthesis [65]; FASN deficiency results in impaired cholesterol retention in the plasma membrane and disrupts Rho GTPase trafficking and JNK activation, which are major mediators of M0 to M1 macrophage transformation [66]. Additionally, FASN can also contribute to the pro-inflammatory role of macrophages by promoting Akt palmitoylation, which further enhances the activation of Akt-MAPK signaling [9]. These studies suggest that FASN plays a crucial role in the polarization and activation of M1-type macrophages (Fig. 3).

Moreover, FASN is also involved in regulating NLRP-3-mediated caspase-1 activation, pro-1β expression, and reactive oxygen species (ROS) production in macrophages [67]. Furthermore, the FASN protein can promote the formation of ox-LDL-induced macrophage foam cells, thereby augment the activation of macrophages and contribute to the pathogenesis of atherosclerosis [68]. However, few studies have been conducted on regulating M2 macrophage polarization by FASN. Thus far, only one study has shown that circ_0018909 induces the polarization of M0 macrophages to M2 macrophages by regulating the signaling axis of miR-545–3p-FASN and then participates in the pathogenesis of pancreatic cancer [54]. It follows that FASN is widely involved in regulating M1-type macrophage polarization.

In addition, FASN is also involved in regulating the function and chemotaxis of macrophages. HIF-1α deletion promotes lipid formation and lipid accumulation in macrophages through the mTOR-SREBP-1c-FASN axis; thus, myeloid cells from myelo-restricted HIF-1α-deficient mice and individuals with HIF1A gene polymorphism dysfunction are more likely to be infected with *Leishmania donovani* through increased lipogenesis, thereby suggesting that FASN-mediated lipogenesis and lipid accumulation are involved in the regulation of the bactericidal function of macrophages [21]. FASN can also contribute to the pro-inflammatory role of macrophages by promote Akt palmitoylation, which further enhance the activation of Akt-MAPK signaling [9]. Furthermore, FASN can also regulate the recruitment and infiltration of macrophages [69, 70]. For instance, FASN deficiency alters membrane order and composition, impairing the retention of plasma membrane cholesterol and disrupting Rho GTPase trafficking-a process needed for cell adhesion, migration and activation. This disruption leads to reduced recruitment of macrophages to adipose tissue and chronic inflammation in mice, preventing diet-induced insulin resistance [66]. Also, LPS can trigger macrophage chemotaxis via the SREBP-1c/FASN/palmetto signaling pathway, inhibition of FASN expression significantly alleviated LPS-augmented CCL2 secretion, indicating that FASN can contribute to the recruitment and progression of macrophages in periapical inflammation [69]. Together, considering the regulatory effect of FASN on macrophages, FASN may become a therapeutic direction for macrophage-mediated inflammatory diseases (Fig. 3).

### T lymphocytes

T cells, which mainly mediate adaptive immunity, are composed of a group of heterogeneous lymphocytes with different functions. CD8^+^ T cells play an important role in the immune system’s defense against pathogens and tumors [71], while CD4^+^ T cells, including subpopulations such as helper T cells 1 (Th1), Th17, regulatory T cells (Tregs) and so on, mainly involved in antimicrobial immunity, autoimmunity, and tumor immunity [72, 73].

Notably, the activation and differentiation of T cells are often accompanied by significant changes in fatty acid metabolism, and FASN-mediated lipid metabolism can widely affect the survival, differentiation, and function of T cells. Notably, one research has shown that FASN is a key metabolic control that generates the inflammatory subgroups of Th17 cells. The activation of Th17 cells by IL-23 and Pam3CSK4, an agonist of TLR2/1, can lead to the increased expression of FASN, subsequently contributing to the survival and proliferation of Th17 cells; the inhibition of FASN, particularly in Th17 cells, significantly attenuated the disease in a mouse model of experimental autoimmune encephalomyelitis; Conversely, the inhibition of FASN function promotes the production of IFN-γ by Th1 and Th1-like Th17 cells [12]. However, the molecular mechanism by which FASN plays a unique role in promoting Th17 differentiation remains to be determined. It is well known that Th17 is differentiated by Th0 cells under the induction of IL-6 and IL-23, which activate the JAK-STAT3 signaling pathway and induce the expression of important transcription factors, such as RORγ and IL17 along with other pro-inflammatory factors [74]. Since targeting FASN had no effect on the expression of other lineage-specific transcription factors, like Foxp3, GATA binding protein 3, and T-bet, FASN may directly or indirectly regulate the activation of JAK-STAT3 signaling pathway or the transcription of the Th17 genetic program. Therefore, future experiments will be necessary to determine the mechanism underlying the regulation of Th17 function by FASN (Fig. 3).

In addition, FASN has been proven to be related to restimulation-induced cell death (RICD). RICD is a type of cell death triggered by T cell receptor (TCR) reinvolvement in activating effector T cells to ensure that the expansion of the T-cell population is maintained in an inhibited state. Blocking FASN with compound C75 can markedly protect CD4^+^ T cells from the effects of RICD, thus suggesting that FASN can promote sensitivity to RICD (Fig. 3) [75].

Regulatory T cells (Tregs) possess immunosuppressive functions and can maintain immune homeostasis, suppress inflammation, and promote cancer [76]. The advantage of Tregs in the TME is closely tied to energy supply pathways associated with lipid metabolism. Notably, FASN-mediated fatty acid synthesis is crucial for the functional maturation of Treg cells. Despite normal TCR-induced proliferation, FASN deletion impaired the TCR-dependent upregulation of Treg activation and maturation markers, including GITR and CD44; moreover, de novo fatty acid synthesis mediated by FASN contributes to the functional maturation of Treg cells, and the loss of FASN from Treg cells inhibits tumor growth, which may be related to dysregulated activation of PI3K in intratumoral Treg cells [5]. These data collectively indicate that FASN contributes to the functional maturation of Treg cells, and inhibiting FASN-dependent lipid synthesis, and the release of metabolic signals from Tregs effectively suppresses the antitumor immune response (Fig. 3) [5]. Another study also found that the upregulation of FASN expression in Tregs of APS-1, an organ-specific autoimmune disease characterized by single-gene mode inheritance, indicates increased metabolic activity and correlates with functional impairments of Tregs [13]. However, the exact mechanism remains unclear. Taken together, the above studies confirm that FASN-mediated metabolic reprogramming enhances the functional specialization of Treg cells in tumors and provides a novel approach for the targeted therapy of Treg cells in tumors.

Moreover, FASN is also involved in regulating the infiltration and activation of T cells. One study showed that the PI3k-α-specific inhibitor CYH33 facilitates the infiltration and activation of T cells by promoting FASN-mediated fatty acid metabolism while weakening the proliferation of M2-like macrophages and Treg cells; this boosts host immunity and inhibits tumor growth [77]. Similarly, another study also reported that FASN expression in gastric cancer tissues was closely related to the levels of immune infiltration of T cells [70]. In conclusion, FASN can regulate the infiltration and activation of T cells (Fig. 3).

### Other cells

As is known, dendritic cells (DCs) play an important role in immune responses by activating naïve T cells [78]. According to previous studies, the synthesis of fatty acids is essential for the maturation and function of DCs. Inhibition of FASN reduced the proportion of CD11c^+^ cells in mouse liver, bone marrow and spleen and improved the ability of DCs to capture antigens [79]. Similarly, FASN is highly expressed in ovarian cancer and can inhibit the capacity of tumor-infiltrating DCs (TIDCs), thereby reducing the ability of T cells to fight tumors (Fig. 3) [80].

Neutrophils, the most important innate immune cells against pathogenic microorganisms, play a promoting role in the pathogenesis of sepsis. The Toll-like receptor (TLR)/myeloid differentiation major reactive protein (MyD88) signaling pathway exacerbates sepsis by affecting the migration of neutrophils to the site of infection. Notably, FASN inhibition by C75 could improve neutrophil chemotaxis and their survival rate in septic mice. C75 specifically blocks the TLR/MyD88 signaling pathway in neutrophils, inhibiting CXCR2 internalization, which in turn promotes the ability of neutrophils to chemotaxis IL-8, thus effectively inhibiting septic inflammation (Fig. 3) [8].

Notably, B cells play a central role in humoral immunity. Germinal centers (GCs) are the sites where B cells differentiate into plasma cells, and produce antibodies; they are therefore essential for humoral immunity [81]. One study has shown that the expression levels of SREBP and FASN in GC B cells are higher than those in naive B cells, and the signaling axis of SREBP-FASN can promote the proliferation of GC B cells through upregulation of lipid homeostasis in vivo (Fig. 3) [82].

## FASN and immune-related diseases

### Tumors

Malignant tumors are a serious risk to people’s health and affect social labor productivity. The immune system has been recognized to play an irreplaceable role in controlling the pathogenesis of tumors. There is increasing evidence that the local tumor microenvironment (TME) has immunosuppressive properties [83]. Previous studies have mainly focused on the function of FASN in the proliferation and metastasis capability of tumor cells [2]. Recently, increasing attention has been given to the effect of abnormal activation of this lipogenesis enzyme on the host antitumor immune environment. It has been shown that FASN and FASN-mediated fatty acid synthesis contribute to the pathogenesis of many different types of tumors by regulating the immunosuppressive properties of the TME. For example, as previously mentioned, the lipid synthesis and metabolic signals controlled by FASN in Treg cells are indispensable for their function and maturation; deleting FASN effectively impairs the function and maturation of Treg cells, thereby resulting in an enhanced antitumor immune response, and inhibition of colorectal cancer development [5].

According to several studies, in 35 different cancers, the higher the level of FASN expression, the less the infiltration of immune cells with antitumor function in the TME [84]. For example, FASN was overexpressed in gastric cancer tissues, and its expression was negatively correlated with the immune cell infiltration levels of CD4^+^ T cells, CD8^+^ T cells, macrophages, neutrophils, and DCs [70]. Additionally, in melanoma patients with FASN mutations, the immune cell infiltration levels of initial B cells, CD4^+^ T cells, cytotoxic cells, effector memory CD4^+^ T cells and DCs were enhanced. In contrast, those of Treg cells and M2 macrophages were reduced, thereby preventing the metastasis and proliferation of melanoma cells [85]. It needs to be pointed out in particular that M2 macrophages share common markers and mechanisms of action with mononuclear myeloid-derived suppressor cells (M-MDSCs), which are a type of suppressive myeloid antigen-presenting cells that have been shown to promote tumor progression and correlate with poor prognosis in cancer patients. For example, both cell populations express arginase-1 (arg-1), inducible nitric oxide synthase (iNOS), and programmed death ligand 1 (PD-L1), and they secrete immune-suppressive cytokines such as IL-10. M2 macrophages express CD115 and interferon regulatory factor 8 (IRF8) more intensely but not S100A9 [86]. Additionally, it has been shown that tumor infiltrating MDSCs can differentiate into M2 macrophages.

Moreover, the abnormal expression of FASN has been proved to be associated with the sensitivity and prognosis of malignant tumors to immunotherapy, especially PD-1 therapy [84]. As is known, PD-1 can negatively regulate the function of T cells, and contribute to the immune escape of tumor cells. Docosahexaenoic acid (DHA), an omega-3 polyunsaturated fatty acid, promotes the degradation of PD-L1 by inhibiting the expression of FASN, and then reverses the PD-L1-mediated immunosuppression, thus inhibiting tumor growth [87]. Another study has also shown that the upregulation of FASN participates in the resistance of lung cancer cells to natural killer cell-mediated cytotoxicity [88]. These findings indicate that FASN holds potential as an effective prognostic indicator and immunotherapeutic target for diverse types of malignant tumors.

In addition, in the tumor immune microenvironment, cells with immunosuppressive functions, such as Treg cells, can promote tumor immune escape by inhibiting the differentiation and function of some pro-inflammatory immune cells, including M1 macrophages, dendritic cells, Th1 cells, CD8^+^ T cells and NK cells [89]. For example, M1 macrophages can express a large number of proinflammatory cytokines, including TNF-α, arginase-1 and iNOS, thereby participating in the Th1 cell-mediated anti-tumor immune response, and recruiting CD8^+^ T cells and NK cells [90]. Since specifically blocking FASN in Treg cells can inhibit the maturation and function of Tregs [5], targeting FASN can eliminate the inhibitory effect of Tregs on the antitumor function of immune cells, leading to the activation of M1 macrophages and CD8^+^ T cells. Thus, targeting FASN can enhance antitumor immunity, and has the potential to be a unique approach to tumor immunotherapy.

**Fig. 4:**
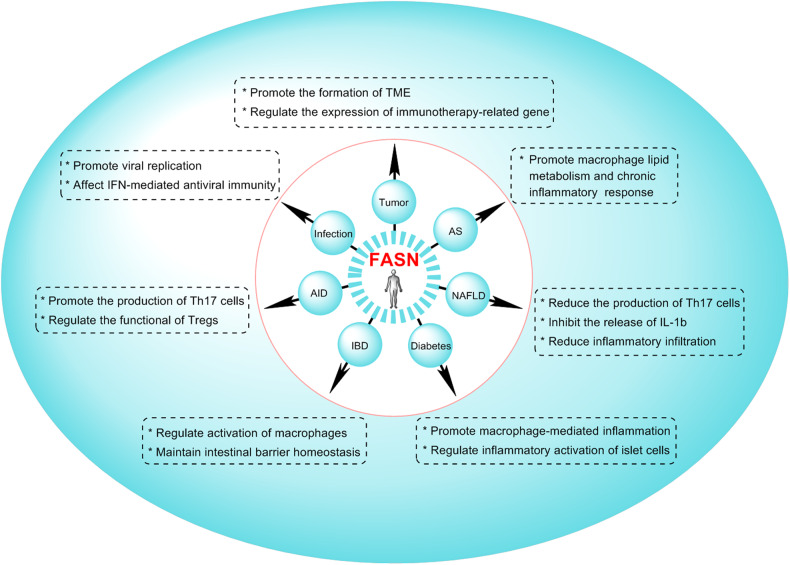
The roles of FASN in the pathogenesis of immune-related diseases. FASN regulates the differentiation, development, chemotaxis, activation, and function of immune cells. AID autoimmune diseases, IBD inflammatory bowel disease, NFLD nonalcoholic fatty liver disease, AS Atherosclerosis.

Taken together, FASN can contribute to the TME formation and tumor immune response. On the one hand, FASN participates in tumor immunity by regulating the infiltration of immune cells in the tumor immune microenvironment; on the other hand, FASN participates in tumor immunity by directly affecting the differentiation and function of immune cells (Fig. 4).

### Infectious diseases

Infectious diseases, such as coronavirus disease 2019 (COVID-19), are local tissue and systemic inflammatory responses caused by pathogen invasion into the body, which can cause multiple organ damage and death and have become a major global public health challenge [91]. It has been confirmed that FASN is involved in regulating the replication of various viruses [92, 93]. For example, the lipid status of cells regulates the replication and infectivity of hepatitis C virus (HCV), and miR-27a can inhibit HCV replication and infection by inhibiting the expression of FASN and SREBP1 [94]; Infection with Singapore Irivirus (SGIV) increases the expression of key enzymes of fatty acid synthesis in vivo and in vitro, such as FASN and SREBP1, and FASN regulates the replication of SGIV by affecting viral gene transcription and protein expression [95]. Regarding COVID-19, cellular lipid synthesis is a prerequisite for SARS-CoV-2 replication. In vitro experimental results and clinical data show that orlistat, a drug inhibitor of FASN, can markedly block coronavirus replication, and renew immune homeostasis [96]. These findings indicate that FASN is involved in regulating virus infection; therefore, FASN inhibitors have promising potential in treating infectious diseases.

Precisely because lipid metabolism regulates viral replication and the host antiviral immune response, the role of lipid metabolism in viral infection has attracted considerable attention. Notably, type I interferons (IFN-I) can trigger a cascade of signals that ultimately activate hundreds of genes known as interferon stimulator genes (ISGs), which together promote an antiviral state. Interestingly, IFN-I can significantly downregulate the expression of FASN, thereby reducing viral infection [10], which reveals a novel mechanism for the antiviral activity of IFN-I, namely the downregulation of metabolic gene expression. Together, FASN can indeed regulate viral replication and host antiviral immune response (Fig. 4).

### Autoimmune diseases

Autoimmune diseases (AIDs) are caused by the abnormal activation of immune cells to imbalance autoimmune homeostasis, and the incidence of these diseases is increasing worldwide [97]. Since FASN has an enormous impact on the activation, differentiation and function of immune cells, it is reasonable to assume that FASN is involved in the occurrence and development of AID. However, to date, few studies have explored the function of FASN in AID.

Multiple sclerosis (MS) is a common inflammatory demyelinating disease, and experimental autoimmune encephalomyelitis (EAE) is a model of MS disease mediated primarily by specifically sensitized CD4^+^ T cells. FASN has been characterized as a key metabolic control for the production of inflammatory subsets of Th17 cells, and inhibition of FASN, particularly in Th17 cells, can reduce the severity of EAE [12].

Rheumatoid arthritis (RA) is a typical chronic systemic autoimmune disease. Morin, a natural flavonoid, can block Th17 differentiation by restricting FASN transcription and subsequently alleviate collagen-induced arthritis. However, another study showed that Bi Zhong Xiao decoction (BZXD) can promote the expression of FASN, and affect fatty acid metabolism, thereby exerting therapeutic effects on RA [98]. It can be seen that the role of FASN in the pathogenesis of RA is still controversial.

Autoimmune polyendocrine syndrome type I (APS-1) is a single-gene mode disease of organ-specific autoimmunity that is caused by mutations in the autoimmune regulatory factor (AIRE) gene. Compared to healthy subjects, FASN expression in Tregs was increased in APS-1 patients; this may be related to the functional defects of Tregs. Functional studies are needed to determine the implications of these findings for APS-1 immunopathogenesis and Treg immunobiology [13]. These studies suggest that FASN truly has a significant regulatory role in the pathogenesis of AID (Fig. 4).

### Inflammatory bowel disease

Inflammatory bowel disease (IBD) is a common disease caused by an overactive immune system that invades the walls of the gastrointestinal tract, thereby causing inflammation and ulcers [99]. It has been shown that FASN contributes to the pathogenesis of IBD: FASN can interact with Hakai and contribute to the occurrence of IBD, suggesting that FASN-mediated intestinal barrier function homeostasis may be one of the important mechanisms in the pathogenesis of IBD [100]. Moreover, the FASN inhibitor C75 can significantly inhibit the expression of FASN and effectively reduce the severity of experimental colitis by inhibiting dextran sodium sulfate (DSS)-induced activation of the inflammatory pathway [101]. Moreover, metformin, a hypoglycemic drug, can improve DSS-induced colitis by blocking the proinflammatory activation of macrophages by inhibiting the FASN/Akt pathway [9]. Thus, FASN participates in the pathogenesis of IBD, and inhibition of FASN may be an effective treatment for IBD (Fig. 4).

### Diabetes

Diabetes mellitus (DM) and its precursor, insulin resistance, is a metabolic disease caused by abnormalities in the transfer, transportation, and/or storage of energy substrates. There is increasing evidence that FASN is associated with DM and insulin resistance. Notably, knocking down FASN expression in macrophages significantly prevents diet-induced insulin resistance and leads to recruitment of macrophages to adipose tissue and chronic inflammatory sites in mice [66], indicating that endogenous fat production in macrophages is essential to develop exogenous adipose-induced insulin resistance.

A major cause of end-stage renal disease is diabetic nephropathy (DN). miR-544 can directly target the mRNA of FASN, and subsequently inhibit glomerulosclerosis and inflammation, thereby alleviating diabetic kidney injury [102], suggesting that FASN is involved in the pathogenesis of DN and that targeting FASN could be a potential approach to treat DN. Also, FASN can regulate the transcription and selective splicing levels of immune/inflammation-related genes in islet cells, thus contributing to the immune metabolism of islet cells [103]. These studies provide new insights for interpreting the function of FASN in the immunological mechanisms underlying the onset of DM and insulin resistance (Fig. 4).

### Nonalcoholic fatty liver disease

The incidence of nonalcoholic fatty liver disease (NAFLD) has remained high throughout the world, and it has become the most common cause of chronic liver disease. However, to date, there is no recognized drug treatment for NAFLD. FASN has become an attractive therapeutic target for NASH due to its ability to aggravate the development of NAFLD by mediating pro-inflammatory and fibrotic signals. The inhibition of FASN activity could alleviate the disease by reducing Th17 cell production and IL-1β release [104]. This finding suggests that FASN inhibition can reduce the inflammation of NAFLD by directly inhibiting the hyperactivation of immune cells.

In view of the crucial role of FASN in aggravating the disease conditions of NAFLD, several studies have conducted targeted FASN therapy for NAFLD and found that the pharmacological inhibition of FASN activity can significantly alleviate NAFLD. For example, some natural plant medicines, such as Schisandrin B, Arteether, and Limonin, can effectively inhibit the activation or infiltration of M1 macrophages, as well as the secretion of proinflammatory factors, by inhibiting FASN-mediated new fat formation and the lipolysis process, thereby exerting a protective effect on NAFLD [105–109]. As is known, M1 macrophages play a critical role in the pathogenesis of NAFLD by expressing high levels of pro-inflammatory cytokines and producing large amounts of reactive oxygen species and nitrogen substances [110]. These actions promote steatosis, inflammation, and hepatocyte damage. Moreover, the activation of macrophages can recruit the accumulation of other nonresident inflammatory cells, including B lymphocytes, T lymphocytes, and neutrophils, which jointly participate in liver inflammation and promote the progression of NAFLD [111]. Thus, FASN inhibition is emerging as an attractive therapeutic potential in the treatment of NAFLD due to targeting macrophages (Fig. 4).

### Atherosclerosis

Atherosclerosis (AS) is a chronic inflammatory disease caused by abnormal accumulation of lipids. Eukaryotic initiation factor 6 (Eif6), a rate-limiting factor for protein translation, can widely affect cell metabolism; deletion of Eif6 can reduce AS by inhibiting FASN and suppressing lipid metabolism in macrophages [59]. In addition, miR-15a-5p can directly target the 3’ UTR of FASN mRNA and restrain the expression of FASN, thereby alleviating the inflammatory response and arterial injury in diabetic AS rats [112]. These studies showed that FASN participates in the pathogenesis of AS (Fig. 4).

## Outstanding questions

Although existing studies have shown that FASN and FASN-mediated fatty acid metabolism participate in regulating the development of a variety of diseases, there are still many gaps in the mechanism of FASN action in immune-related diseases that need to be studied. Available data indicate that targeting and inhibiting FASN activity holds great promise in treating immune-associated diseases, but a great deal of work remains to be done to clarify the uncertainties that may arise from targeted inhibition of FASN. At the same time, further mechanistic studies are needed to determine the specific function of FASN and FASN-mediated fatty acid synthesis in regulating the immune microenvironment to reveal more therapeutic targets and deepen our understanding of the nosogenesis of immune-related diseases.

## Conclusion and perspective

Disrupted fatty acid metabolism is closely related to the pathogenesis of numerous diseases. FASN exerts regulatory control over immune cell survival, activation, differentiation, and function. Consequently, it contributes to the onset and progression of various conditions, including tumors, cardiovascular diseases, inflammatory diseases, autoimmune diseases, infectious diseases, and other pathological states. Moreover, FASN is becoming a potential target for various diseases. Therefore, on the one hand, we need to further clarify the specific mechanisms by which FASN promotes or alleviates the pathogenesis of immune-related diseases by regulating the function of immune cells. On the other hand, targeting FASN to treat immune-related diseases still requires more clinical data. Therefore, developing safer, more economical, and more efficient drugs has become a research hotspot.

### Supplementary information

reproducibility checklist
